# Case Series on the Long-Term Effect of Three Different Types of Maxillary Implant-Supported Overdentures on Clinical Outcomes and Complications

**DOI:** 10.3390/jcm11082251

**Published:** 2022-04-18

**Authors:** Emitis Natali Naeini, Hugo De Bruyn, Ewald M. Bronkhorst, Jan D’haese

**Affiliations:** 1 Department of Dentistry, Radboud University Medical Centre, 6525 GA Nijmegen, The Netherlands; hugo.debruyn@radboudumc.nl (H.D.B.); ewald.bronkhorst@radboudumc.nl (E.M.B.); jan.dhaese@radboudumc.nl (J.D.); 2 Department of Periodontology and Oral Implantology, Faculty of Medicine and Health Sciences, University of Ghent, 9000 Gent, Belgium

**Keywords:** dental implants, maxillary overdenture, prosthodontics, clinical outcome, peri-implantitis

## Abstract

(1) Long-term data on maxillary implant overdentures (IODs) are scarce. This case series evaluated three types of IODs supported by six, four or three implants (Anyridge^®^, Mega’Gen Implant Co., Ltd., Daegu, South-Korea), after 3–5 years in function. (2) A total of 31 patients, with 132 implants, were non-randomly allocated based on available bone or financial limitations. IOD-6 received a telescopic overdenture; IOD-4 a bar; and IOD-3, non-connected implants with locator abutments. Implant survival, bone level changes, probing pocket depth (PPD), plaque index, bleeding on probing (BOP), and technical, biological and aesthetic complications were registered. Impact of suprastructures on bone loss and PPD was analyzed using mixed-effect linear regression models. Differences between groups were analyzed using the ANOVA test for BOP, and Kruskal Wallis test for complications. (3) In total, 23 patients participated in the follow-up (9 female, 14 male), with average age of 62.2 years; 7, 11 and 5 patients in IOD-6, IOD-4 and IOD-3, respectively. Implant survival after 4.4 years on average, was 98% in total; 100%, 97.8% and 93.3% for IOD-6, IOD-4 and IOD-3, respectively. Mean bone loss corresponded to 0.68 mm (SD 1.06, range −4.57–1.51), 0.39 mm (SD 1.06, range −3.6–2.43), and 1.42 mm (SD 1.68, range −5.11–0.74) for IOD-6, IOD-4 and IOD-3, respectively. A statistically significant difference was seen in bone level when comparing IOD-6 to IOD-3 (*p* = 0.044), and IOD-4 to IOD-3 (*p* = 0.018). Mean PPD was 3.8 mm (SD: 0.69; range 2.5–5.3), 3.5 mm (SD 0.59; range 2.33–5), and 3.2 mm (SD 0.56; range 2–4) for IOD-6, IOD-4 and IOD-3, respectively, and differed significantly between IOD-6 and IOD-3 (*p* = 0.029). Incidence of peri-implantitis was 1%. No differences were seen for complications between groups. (4) Maxillary IOD supported by four to six implants is the most reliable treatment regarding implant survival and peri-implant health. More research is needed in the clinical outcomes, in particular the peri-implant health, and complications of maxillary IODs, especially with a reduced number of implants.

## 1. Introduction

For over a century, the standard of care for edentulous patients has been a conventional complete removable denture. This allowed edentulous patients to eat, speak and function again, albeit often with issues of reduced comfort and impaired well-being due to lack of stability or retention of the denture. As the alveolar ridge reduces over time, this results in social, psychological and functional disabilities [1,2]. The use of dental implants for oral rehabilitation has become a highly predictable treatment for fully and partially edentulous patients. According to the literature, good short- and long-term results have been reported for various treatment indications with an implant survival that ranges from 82–98% [1,2,3,4]. However, it has often been the case that implants in the maxilla have a lower success rate than in the mandible [5]. Nowadays, the use of overdentures retained on two implants (IOD-2) has, especially in the mandible, become a reliable and often standardized treatment option for improving retention [2]. Given the historically lower implant survival in the maxilla, a full-arch fixed prosthesis (FAFDP on four to six implants, is the standard of care with a 15-year implant survival of 90.9% [6]). From a cost–benefit point of view, this is a costly treatment, not affordable to many patients. Hence, cheaper solutions for the maxilla have been introduced. The all-on-four concept, using a fixed 10-unit bridge on four implants, yielded survival of 98% after 5 years [7].

Implant treatment success is predominantly based on the stability of the bone surrounding the implant. According to Ästrand et al. [8], major changes in peri-implant bone level can take place between implant placement and prosthetic loading. This peri-implant bone remodeling occurs in order to re-establish the biological width, especially in patients or at sites with thin, soft tissues [9]. This early remodeling is dependent on multiple factors: type of implant used, surgery and prosthetic aspects. Bone loss in the long term is most often a consequence of peri-implant diseases, also inducing pocket formation and suppuration. This, too, is dependent on several factors, including patient genetic disorders, patient smoking and biomechanical factors such as cement or impression material remnants in the peri-implant sulcus [10]; bacterial contamination of the implant components in the presence of a microgap at the interface between the fixture and abutment [11]; and technical issues such as loose screws, mobile components and fractured materials [10]. Cement remnants in the soft tissues is an example of problems associated with clinical handling. Cement remnants can lead to marginal bone resorption and infection due to a foreign body reaction that may also be linked to the leakage of titanium, which is inevitable around oral implants [10]. However, a recent 10-year retrospective study compared the long-term survival and complication rate of screw-retained vs. cement-retained single implant crowns, and found mean bone loss to be significantly greater for the screw-retained group [12].

Peri-implantitis is defined as a plaque-associated pathological condition occurring in tissues around dental implants, characterized by inflammation in the peri-implant mucosa and subsequent progressive loss of supporting bone [13]. According to the 2017 Consensus report of the World Workshop on the Classification of Periodontal and Peri-Implant Diseases and Conditions [13], the diagnosis of peri-implantitis requires the presence of bleeding and/or suppuration, increased probing depths compared to previous examinations and crestal bone loss beyond normal initial bone remodeling [13]. In the absence of previous examination data, peri-implantitis should then be defined by the presence of bleeding and/or suppuration, in combination with probing depths ≥6 mm, and bone levels ≥3 mm apical to the most coronal portion of the intraosseous part of the implant [13]. The prevalence of peri-implantitis varies significantly among studies due to inconsistent definitions, various reporting methods and different study characteristics. A recent critical review reported that the prevalence of peri-implantitis, after a mean follow-up time of 5 years, was between 0% and 39.7%, based on 15 different case definitions [14].

In terms of long-term complications of IODs, veneer fractures are reported to be the most frequent technical complication during a follow-up period of 5 to 15 years, followed by abutment screw loosening/fractures and framework fractures [6]. Biological complications are not always reported and if they are mentioned in studies, they are described generally as ‘soft-tissue inflammation’ [15]. Most of the time, a high level of patient-centered outcomes have been observed [16]. Apart from financial restrictions, other limitations such as insufficient bone volume or an unfavorable jaw relation can sometimes prevent the placement of a sufficient number of implants required for a fixed prosthesis [17,18]. Therefore, an IOD, retained on a reduced number of implants, could be a viable alternative for edentulous patients with compromised oral function. Maxillary IODs have previously been reported to have a relatively high rate of implant loss compared to other implant treatment modalities [17,19]. Implant number, length and inclination may be compromised by anatomic limitations and bone morphology [20]. Moreover, controversy persists regarding factors critical for implant and prosthetic success [21]. The number of implants in a maxillary IOD affects the survival rate, due to the forces on the overdenture being distributed by the bone surrounding the implants. Thus, the more implants, the more evenly the forces are distributed [22]. There are no specific guidelines concerning the number of implants necessary to retain a maxillary overdenture [23]. Bergendal et al. [17] compared two IODs in both the maxilla and the mandible and described a cumulative implant survival rate of 75.4% and 100% respectively, after 7 years in function; it is obvious that this IOD-2 yields too many failures and complications to be advisable. On the other hand, for an IOD without palatal coverage, a minimum of four implants is favorable [21,24,25,26]. These recommendations are further supported by recent studies by Doornewaard et al. [27], who reported 96% implant survival up to 4 years, for maxillary IODs retained on a bar on four implants.

Currently, long-term data on maxillary IODs with various designs of retention are scarce. The aim of the present study was to evaluate the effect of three different types of maxillary overdentures on the clinical outcome, including survival, peri-implant bone loss, peri-implant health and complications of implants in the edentulous maxilla, after 3–5 years in function.

## 2. Materials and Methods

### 2.1. Patient Selection and Treatment Allocation

Originally, 31 referred patients presenting with a complaint of lack of retention in their removable maxillary denture, were included in this clinical case series, all of whom had a sufficient amount of bone to install at least 3 implants as confirmed on CBCT. The patients were in good health. If necessary, patients were scheduled for periodontal treatment of the residual mandibular teeth prior to implant placement. Smokers up to 10 cigarettes/day were also included. Patients suffering from severe osteoporosis and/or non-controlled diabetes mellitus or taking anticoagulants that could not easily be substituted were also excluded. A healing period of approximately 2 months after tooth extraction was allowed before implant surgery. Hence, initial post-extraction bone resorption occurred before surgery. This study used a practice-based approach and group allocation was not randomized. Patients were allocated into one of the 3 treatment groups based on the availability of bone on the CBCT firstly, and secondly, based on their financial possibility. So, in the case of sufficient volume of bone on the CBCT, the patient was advised to receive 6 implants and restored with a type of telescopic overdenture (IOD-6). Where there was a lack of bone volume, or financial restrictions from the patient, 4 implants were advised to support a bar-retained overdenture (IOD-4). As the last option, 3 non-splinted implants were placed, each with a locator abutment to retain the overdenture (IOD-3). All patients signed a written consent before treatment and approval was obtained from the ethical committee of the Ghent University hospital (ID B670201420643).

### 2.2. Surgical and Prosthetic Procedure

The implants used in this case series (Anyridge^®^, Mega’Gen Implant Co. Ltd., Daegu, Korea) had a width varying from 3.5 to 5.5 mm and a length varying from 7 to 13 mm and a 5° morse taper internal connection. It has a moderately rough surface with an average S_a_ value of 1.3 μm and a 0.8 mm thread pitch. All the implants were placed using a conventional one-stage surgical approach by the same periodontist (JD). Following local anaesthesia, a crestal incision was made to elevate the full-thickness mucoperiostal flap and expose the bone. Using a duplicate of the pre-existing denture as a surgical guide plate (with palatal support), the osteotomies were prepared at 1500 rpm with abundant irrigation. All implants were planned to be placed equicrestally or slightly subcrestal, using a maximum insertion torque of 35 Ncm. Healing abutments were connected using hand torque (±15 Ncm), prior to flap closure. Peri-apical intraoral radiographs were obtained by the surgeon immediately after implant placement (baseline), with commercially available film holders using the parallel long-cone technique in order to visualize the implant threads and alveolar bone level. A conventional relined complete denture served as a provisional solution. A prescription was given for Ibuprofen 600 mg (3 times a day, for as long as necessary), Amoxicillin 1 g (2 times a day for 5 days) and 0.12% chlorhexidine mouth rinse (2 times a day, for 2 weeks). One week after surgery, patients returned for suture removal, and afterwards they returned for regular post-operative maintenance, including relining of the denture if necessary.

**Figure 1:**
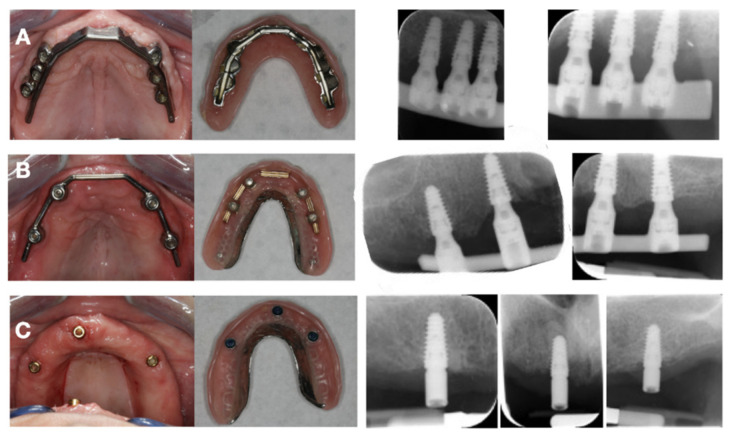
Restorative procedure for group IOD-6 (**A**), IOD-4 (**B**) and IOD-3 (**C**), showing the intra-oral implant position and suprastructures 3 months after placement, final prosthesis and periapical radiographs obtained after the placement of final prosthesis.

Three months after surgery, the stability of the implants was clinically evaluated. A first impression was made using an irreversible hydrocolloid material (Cavex, Haarlem, The Netherlands). Based on this impression, an individual open tray was made and an impression on abutment ((IOD-6, IOD-4) or implant (IOD-3) level was obtained using a polyether impression material (Impregum, 3 M, ESPE, St. Paul, MN, USA). After a screw-retained jaw registration, try-in and additional fit of the metallic structure (for IOD-6 and IOD-4), placement of the final prosthesis was carried out. During placement of the final restoration the occlusion was carefully checked and hygiene instructions were given to obtain good oral hygiene maintenance. Figure 1 shows the intra-oral implant position and suprastructures for each group, 3 months after placement.

### 2.3. Clinical Assessment

**Figure 2:**
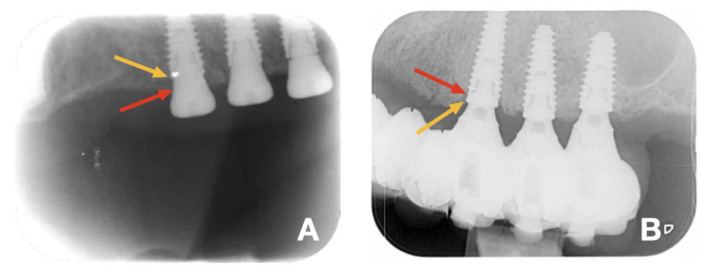
Radiograph obtained at the day of implant placement (**A**), and after 3–5 years of function (**B**). The yellow arrow indicates the reference point located at implant–abutment interface; the red arrow indicates the bone-to-implant contact level.

Patients were assessed at the implant center after 6 months and 1 year, after which they returned to their referring dentist for regular maintenance. As part of this research investigation, the originally treated patients were invited to participate in a clinical research investigation after at least 3 years in function. An external periodontal specialist (ENN) from the University of Ghent, not involved in the original surgical interventions, evaluated all the listed clinical and radiographic parameters. The peri-implant health was evaluated using the following parameters: probing pockets depth (PPD), plaque score using the Silness—Loë plaque index, and bleeding on probing (BoP) using the modified bleeding index (mBI) by Mombelli et al. [28]. In this study, the periodontal parameters were later dichotomized into a variable of 1 or 0, so that when an implant presented with any bleeding, it was subsequently given a score of 1, and 0 when no bleeding was observed. This score was then divided by the number of implants to give the relative number of implants with bleeding per patient. An implant was classified as a failure when it was removed because of mobility, loss of integration, ongoing bone loss, signs of infection and/or patient discomfort [29]. Early failures (prior to loading) have been excluded from this study. Baseline peri-apical radiographic exposures were obtained immediately after implant insertion. The bone level was measured per treatment group, at baseline and at the time of scientific recall (3–5 years after implant surgery). The software used was AxioVision Rel. 4.8 (Carl Zeiss MicroImaging GmbH, Germany), using an accuracy of 0.01 mm. The known distance between the implant thread (0.8 mm) was used for the calibration of the radiographs. The implant–abutment connection was determined as the baseline reference point (0 mm) from which point the closest bone implant contact was measured (Figure 2). Both mesial and distal bone level was measured from the implant.

Complications were divided into technical (implant fracture, screw/abutment fracture, screw/abutment loosening, loss of retention), aesthetic (fracture of veneering material/framework of prosthesis) and biological (mucositis, peri-implantitis).

### 2.4. Statistical Analysis

Implant number was used as the unit for the statistical analysis on bone loss, PPD and BoP. The impact of different suprastructures on bone loss and PPD was analyzed using mixed-effect linear regression models that accounted for the clustering of implants per patient by incorporating a random intercept for variable patient. In both analyses, the baseline measurement was included in the model as independent variable, together with the variable suprastructure, using IOD-6 as a reference group. These two analyses were performed with the library lme4 in R version 3.6.2. The comparison of the scoring of bleeding upon probing between the suprastructures was carried out by means of an ANOVA test, while the comparisons of the complications per group was done with the Kruskal Wallis test. The ANOVA and the Kruskal Wallis tests were carried out using IBM**^®^**SPSS**^®^** 25.0 (SPSS Inc., Chicago, IL, USA).

## 3. Results

### 3.1. Clinical Outcome

A total of 132 maxillary implants were originally inserted in 31 patients (17 male, 14 female); 3 implants failed prior to loading and were replaced, and 1 failed after loading; 23 patients (102 implants; 62.2 years (SD 6.9, range 48–77) participated in the follow-up study (9 female, 14 male). Of the eight patients who dropped out, five were untraceable, two refused to participate and one was unable to attend due to illness. One patient originally selected for IOD-6, requested a bar-retained overdenture be made, and was later allocated to prosthetic group IOD-4. The mean follow-up time was 4.4 years (range 3.4–5.1), and comparable between groups. The age of the subjects was comparable between groups as well. With the limitation of the dropouts (8/31) for which no information is available, the current overall survival rate was 98% for the whole patient group, and 100%, 97.8% and 93.3% for IOD-6, IOD-4 and IOD-3, respectively. Table 1 summarizes the descriptive results for clinical outcomes at baseline and at time of follow-up. The fraction of bleeding implants for IOD-6, IOD-4 and IOD-3 were 78.6%, 54.5% and 53.3%, respectively (*p* = 0.229).

### 3.2. Bone Level and Probing Pocket Depth

**Figure 3:**
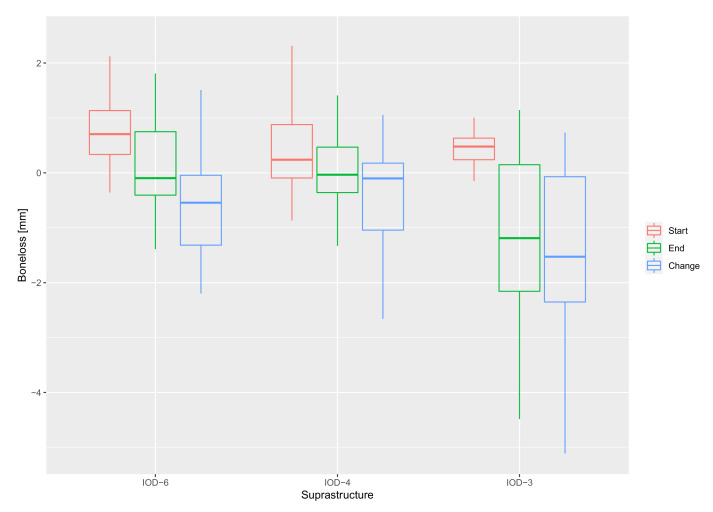
Boxplot of the median bone level in mm at baseline (start) and after 3–5 years (end) for all 3 treatment groups, and the overall bone loss (change).

The effect of the suprastructure on bone loss was calculated by using the bone level at baseline and the suprastructure as independent variables. IOD-6 was used as the reference for the mixed model analysis, as shown in Table 2. No statistically significant difference was seen when comparing IOD-6 and IOD-4 (*p* = 0.828). However, a statistically significant difference was seen when comparing IOD-6 and IOD-3 (*p* = 0.044). Initially, the mixed model analysis only used IOD-6 as the reference; however, to give more clarity to the reader, IOD-4 was also compared with IOD-3 and a statistically significant difference was seen here as well (*p* = 0.018). Hence, IOD-3 shows more bone loss as compared to the other two treatment groups. Figure 3 shows a boxplot of the bone level at baseline and after 3–5 years, and the overall bone loss (mm) over time for all three treatment groups. With regards to other clinical parameters, there was a statistically significant difference in probing pocket depths between IOD-6 and IOD-3 (*p* = 0.029).

### 3.3. Peri-Implant Health

The incidence of peri-implantitis in the study population is 1% according to the 2017 The consensus report [13], as one of the implants showed bone levels of 3 mm apical to the most coronal portion of the intraosseous part of the implant, as well as a probing depth of >5 mm combined with bleeding on probing, as shown in Table 3.

### 3.4. Complications

Table 4 shows each complication encountered per patient, per treatment group, from the time of implant placement to the time of follow-up. For six patients in IOD-6, a technical complication occurred. In these cases, the patients incurred a loss of retention rubbers (resolved by changing the retention rubbers), and a crown fracture (patients 5 and 6). One patient experienced a biological complication, peri-implantitis, and was treated with antibiotics (patient 1). For IOD-4, 1 patient experienced a biological complication whereby the implant had not osseointegrated (patient 12). This implant was removed and a new implant replaced it. Four patients experienced aesthetic complications in wear of the IOD. No intervention was carried out for these patients. Two biological complications were reported in group IOD-3; one patient had peri-implantitis and was treated by carrying out flap surgery (patient 19); another patient experienced late implant failure and the implant was removed (patient 23). Three patients experienced technical complications, including prosthetic fracture (patients 20, 21, 23), and had new prostheses made. Loss of retention rubbers were also reported and replaced (patients 21 and 23). The Kruskal Wallis test was used for analysis between groups with an uneven distribution; there was no statistically significant difference.

## 4. Discussion

Currently there is no consensus regarding the number of implants required for an implant-supported maxillary overdenture. The present study assessed the clinical outcomes and complications with three types of IODs retained on three, four or six implants, after 3 to 5 years of function.

### 4.1. Survival

Three implants failed before functional loading and were replaced. Uncontrolled premature contacts of the healing abutments with the provisionally relined prosthesis could be a possible reason for this failure. Although there is no evidence for the suggestion that bruxism may cause an overload on dental implants, this could be a plausible explanation for the fact that the other implant failed after functional loading of an overdenture with a locator system, in a patient with bruxism. However, it remains difficult to draw strong conclusions regarding the cause of implant failure. In the present study, two implants failed after loading, resulting in an overall survival rate of 98% after a mean follow-up period of 4.4 years. This is in accordance with a systematic review by Slot et al. [22], where survival rates of >95% were reported. Implant survival for IOD-6, IOD-4 and IOD-3 was 100%, 97.8% and 93.3%, respectively, suggesting an intense relation between number of implants and risk of failure. A possible explanation could be the design of the anchorage system. Meijer et al. [30] found that in the case of a bar structure between the implants, the force distribution is spread to the bone surrounding the bar on two implants, whereas when solitary ball attachments are loaded, the forces are distributed to the surrounding bone of a single implant. Raghoebar et al. [31] described in a systematic review an implant survival rate of 98.1% after 1 year in a case of six or more implants and a splinted anchorage. In a case of less than four implants and a splinted anchorage or a non-splinted anchorage, an implant survival rate of 97.0% or 88.9% was observed, respectively. They concluded that an implant-supported (less than four implants) maxillary overdenture provided with a splinted anchorage is accompanied with a high implant survival rate, while there is an increased risk of implant loss when less than four implants with a non-splinted anchorage are used. This is in accordance with the present study. Higher survival rates of 100% and 99.2% were reported in another RCT comparing four and six implants, respectively, supporting splinted maxillary overdentures after an observation period of 5 years [32]. It can be argued that in this RCT, all included patients initially had enough bone to receive six implants; however, for the purpose of the study, some received only four [32]. The current study is not only not an RCT, but uses a practice-based approach, which describes realistic situations whereby patients are treated with fewer implants due to not having enough bone volume to being with. Hence the high failure rate with reduced number of implants may also reflect the more critical bone condition of the treated patients in the locator-retained group (IOD-3). Conversely, lower survival rates of 82.3% were found in a prospective study evaluating free-handed flaplessly placed one-piece maxillary mini dental implants with ball attachments, supporting a metal-reinforced, horse-shoe removable denture, after just 2 years in function [33]. In these cases, the patients had reduced bone volume to begin with and could not be treated with conventional implant diameters [33]. A drawback of the present study is the small sample size, due to the study site being a referral center whereby general dentists would refer the patient back to the surgeon only when issues occurred.

### 4.2. Bone Loss

Between baseline (i.e., implant insertion) and an average of 4.4 years in function, a mean bone loss of 0.65 mm was observed for the whole patient group, and 0.68 mm, 0.39 mm and 1.42 mm for IOD-6, IOD-4 and IOD-3, respectively. Bone loss around implants can occur for several reasons, including as a consequence of initial bone remodeling [8] and of peri-implant disease. In this study, we controlled certain co-factors, which could affect the bone level; implant type and implant surgery was comparable for the three treatment modalities and all IODs were screw-retained. The latter may explain the steady state in bone level over time and only 1% incidence of peri-implantitis. The reported bone loss between implant insertion and long-term follow-up in this study includes the initial bone remodeling and additional bone loss after functional loading. This may therefore cause an overestimation in comparison with many clinical studies that do not take initial bone remodeling into account because they consider the time of loading, being many months after bone healing took place, as baseline for bone loss calculation. One notable finding in this study is the statistically significant effect of the suprastructure on the bone levels over time, namely when comparing non-splinted, locator attachment–supported overdentures (IOD-3) to the splinted alternatives (IOD-4 and IOD-6). A mean bone loss of 1.42 mm was reported for IOD-3. This significant difference could be related to the movement of the denture caused by having only three implants with locator attachments. Pressure of the denture on the mucosa, in combination with uneven force distribution, could explain this, as also suggested by Kamei et al. [34]. The latter used finite element analysis and found that maxillary denture retained on four implants results in less displacement than in models with fewer implants. Stress is also reduced when implants are inserted in the premolar area [34]. Similar conclusions were drawn from a study carried out by Dimililer et al. [35], aiming to find the optimal implant location, number and diameter to support a maxillary implant-supported overdenture. The implant diameter had no significant effect on stresses; however, as the number of implants increased, decreased stress values were observed in the peri-implant bone and implants [35]. It is well established that using four implants to support a more rigid suprastructure, may overcome this possible loading effect [31,34,35]. Finally, another reason could be the lack of splinting in the IOD-3 system, although it is interesting to see that in a recent systematic review [36] comparing splinted vs. unsplinted designs for a maxillary overdenture supported by four implants, no statistical difference was detected in the survival rate of implants between the splinted implant group and the unsplinted implant group. It can be disputed that this systematic review included many RCT’s whereby implants were placed in patients who had sufficient bone volume.

### 4.3. Peri-Implant Health

In the current study, peri-implant bone loss was not influenced by plaque and bleeding. This corroborates with studies that have also found no relation between the probing depth or bleeding and the mean bone loss, mean follow-up time, and reported prevalence of peri-implantitis [14,37]. Verhoeven et al. [38] reported a rather poor specificity and sensitivity for the plaque and bleeding index and considered these periodontal parameters as unreliable for clinical evaluation in implant dentistry. They suggest that radiographs remain the most important source of information to assess peri-implant bone level changes around dental implants. Generally speaking, the mean pocket depth for this study was low as compared to other studies [14]. A statistically significant difference was seen between IOD-6 and IOD-3 (*p* = 0.029). IOD-6 showed a higher relative number of implants showing mucositis as compared to IOD-4 and IOD-3. The total score for the total patient group was 62%. Despite IOD-6 showing a higher fraction of bleeding implants in this study, the Anova test showed no statistically significant difference when comparing the effect of suprastructure on bleeding on probing (*p* = 0.229). A plausible reason for this could be that IOD-6 has more implants than other groups, and so a higher relative bleeding score is to be expected, as well as increased difficulty maintaining oral hygiene the more implants there are. Currently, literature reporting on the epidemiology as opposed to patient-related outcomes and prosthetic complications of peri-implant diseases in this specific group of patients is scarce. Onclin et al. [39] reported patient-level incidence of peri-implant mucositis of 37.7% after 5 years and 64.6% after 10 years. When peri-implantitis is defined according to the aforementioned criteria [13], then one implant was reported as having peri-implantitis in this study. In a recent review [19], the prevalence of peri-implantitis on implant level ranged from 0–40%. The definition of peri-implantitis varies considerably between studies, mostly due to dissimilar thresholds for bone loss. This obviously makes comparisons between studies difficult. For example, Meyle et al. [40] reported a high prevalence of 24% after 10 years. The threshold for bone loss for the diagnosis for peri-implantitis was ‘any bone loss’, which explains the high reported prevalence. If one were to apply the guidelines of the 8th European Workshop on Periodontology on their material, the prevalence would be 0%.

### 4.4. Complications

There was no statistically significant difference in the effect of the suprastructures on complications experienced. Each IOD group reported technical, biological and aesthetic complications, which is comparable to what is reported in the literature; maxillary IODs are described to have a relatively high number of complications in general, especially during the first year in function [21], with both technical and biological problems being observed. The majority of complications are related to the weakness of the anchorage components connecting bar and overdenture [41] or loosening and fracturing of the retention system [21]. Conversely, Slot et al. [32] reported that prosthetic complications were scarce, and only restricted to the repair of denture base and teeth when an IOD with built-in cobalt chromium reinforcement structure and gold retentive clips was used. This would explain the minimal prosthetic complications [42]. In this study, financial limitations of the patients meant that such reinforcements could not be provided, and a simpler prothesis was created.

### 4.5. Limitations

One drawback of the present study is the small sample size. This is due to the study being private practice-based research, with no financial compensation for patients taking part in the study. The same can be said for the number of drop-outs, as the research is based on referred patients, which makes long-term follow-up more difficult. For the same reason, one can only analyze the baseline radiographs available; therefore, not being able to guarantee that the follow-up radiographs would be obtained at the same angulation as baseline, which could have limited the study. However, only radiographs whereby the threads of the implants were clearly visible were analyzed, and calibration was always carried out using the thread pitch of the implant. Furthermore, peri-implant bone loss is often described as a circumferential disease, suggesting that the direction of the beam on the implant does not alter the result [43]. The lack of control in the condition of the antagonistic jaw was also a limitation, due to the limited number of cases. On the other hand, the denture design was not different in terms of occlusion and articulation pattern, and the prosthodontist strived to attain a balanced occlusion and articulation, thereby equally distributing the load on the dental implants.

## 5. Conclusions

A maxillary overdenture supported by four to six implants is a reliable treatment regarding survival of the implants and peri-implant health. Within the aforementioned limitations of the study, there was no clinical difference in IODs supported by four or six implants, which confirms the existing evidence. More bone loss was observed when only three non-splinted implants support a maxillary overdenture, suggesting possible overloading. However, longer follow-up time is needed to assess whether this reaches a stable situation or instead leads to future complications such as ongoing bone loss, resulting in peri-implantitis or even implant loss. More research is needed in terms of complications, patient-centered outcomes and value-based care with various types of maxillary implant overdentures.

## Figures and Tables

**Table 1:** Table showing descriptive results of the population and clinical outcomes at baseline and at time of follow-up.

		Total Patient Group	IOD-6	IOD-4	IOD-3
Number of patients	Baseline	31	9	12	10
	Follow-up	23	7 (−1 §)	11 (10 + 1 §)	5
Number of implants	Baseline	132	54	48	30
	Follow-up	102	42	46 (44 + 2 §)	15
Bone level at baseline (mm)	Mean (SD; range)	0.55 (0.74; −1.06–2.48)	0.70 (0.77; −1.06–2.13)	0.45 (0.77; −0.87–2.48)	0.39 (0.39; −0.50–1.01)
Bone level at follow-up (mm)	Mean (SD; range)	−0.10 (1.01; −4.49–2.14)	0.02 (0.91; −2.8–1.81)	0.06 (0.70; −1.33–2.14)	−1.03 (1.61; −4.49–1.15)
Bone loss (mm)	Mean (SD; range)	0.65 (1.20; −2.44–5.11)	0.68 (1.06; −1.51–4.57)	0.39 (1.06; −2.43–3.6)	1.43 (1.68; −0.74–5.11)
PPD (mm)	Mean (SD; range)	3.6 (0.66; 2.0–5.3)	3.8 (0.69; 2.5–5.3)	3.5 (0.59; 2.33–5)	3.2 (0.56; 2–4)
BOP (relative %)	Mean (SD; range)	61.6% (31.1%; 0–100%)	78.6% (24.9%; 33.0–100%)	54.5% (33.2%; 0–100%)	53.3% (29.8%; 33.0–100%)

(§) represents 1 patient originally selected for IOD-6 switched to an overdenture on a bar on 6, instead of 4 implants.

**Table 2:** Table demonstrating the effect of suprastructure on bone loss and probing pocket depth (PPD). IOD-6 used as the reference for the mixed model analysis. When comparing IOD-6 to IOD-3, a statistically significant difference was seen for the effect of the suprastructure on the bone level (*p* = 0.044), as well as on PPD (*p* = 0.029).

	Variable	Effect	95% CI	*p*-Value
Bone Loss (mm)	Intercept	−0.030	[−0.642 … 0.583]	0.925
	Bone level at baseline	0.074	[−0.142 … 0.290]	0.503
	IOD-6 vs. IOD-4	0.085	[−0.685 … 0.855]	0.828
	IOD-6 vs. IOD-3	−0.985	[−1.940 … −0.028]	0.044
	IOD-4 vs. IOD-3 *	−1.070	[−1.959 … −0.180]	0.018
PPD (mm)	Intercept	3.754	[3.427 … 4.080]	<0.001
	IOD-6 vs. IOD-4	0.188	[−0.612 … 0.237]	0.387
	IOD-6 vs. IOD-3	−0.599	[−1.136 … −0.062]	0.029
	IOD-4 vs. IOD-3 *	−0.411	[−0.917 … −0.094]	0.111

* Additional comparison has been added for clarity to the reader to see the difference between these groups as well.

**Table 3:** Table showing number of implants with corresponding bone loss (mm) and probing pocket depth (mm). The number in brackets is the number of implants that showed bleeding on probing, followed by the percentage of implants which showed bleeding out of the total number of implants (n = 102). Only one of the implants was classified as having peri-implantitis, denoted in bold.

	Pocket Depth				
Bone Loss	≤3 mm	>3 mm and ≤4 mm	>4 mm and ≤5 mm	>5 mm	Total
<0 mm	5 (2,1.9%)	23 (12, 11.8%)	4 (3, 2.9%)	1 (1, 0.9%)	33
≤1 mm	8 (4, 3.9%)	23 (17, 16.7%)	5 (4, 3.9%)	0	36
>1 ≤2 mm	7 (4, 3.9%)	9 (7, 6.9%)	3 (3, 2.9%)	0	19
>2 ≤3 mm	2 (0)	9 (5, 4.9%)	2 (2, 1.9%)	**1** **(1, 0.9%)**	14
Total	22 (10, 10.2%)	64 (41, 41.8%)	14 (12, 12.2%)	2 (2, 1.9%)	102

**Table 4:** Table showing distribution of all complications encountered per patient, per treatment group, at the time of follow-up, and corresponding mean bone loss (a negative number indicates bone gain) and probing pocket depth (PPD).

				Complications			
Suprastructure	Patient ID	Total No.	Technical	Biological	Aesthetic	Mean Bone Loss (mm)	Mean PPD (mm)
IOD-6	1	1		1		1.84	4.42
	2	2	2			0.60	3.69
	3	2	2			−0.06	3.86
	4	4	4			0.72	3.03
	5	1	1			0.77	3.53
	6	2	1			0.25	3.14
	7	1	1			0.61	4.61
IOD-4	8					0.98	2.92
	9	2			2	−0.01	3.58
	10	1	1			0.16	4.08
	11					1.15	3.38
	12	2		1 *	1	−1.05	3.88
	13					0.14	4.29
	14	1			1	0.64	3.75
	15					−0.20	3.42
	16	1			1	0.18	3.38
	17					2.10	3.21
	18					−0.12	3.42
IOD-3	19	1		1		3.49	3.44
	20	1	1			1.83	3.33
	21	4	4			−0.67	3.11
	22					1.91	2.72
	23	3	2	1 **		0.20	3.17

* Implant lost before functional loading and replaced. ** Implant lost during follow-up.

## Data Availability

The data presented in this study are available on request from the corresponding author. The data are not publicly available due to ethical restrictions.
